# The efficacy of upfront craniocerebral radiotherapy and epidermal growth factor receptor-tyrosine kinase inhibitors in patients with epidermal growth factor receptor-positive non-small cell lung cancer with brain metastases

**DOI:** 10.3389/fonc.2023.1259880

**Published:** 2024-01-19

**Authors:** Jianxi Zhou, Yingnan Zhou, Yunchuan Sun, Li Xiao, Hongling Lu, Xiaoming Yin, Kui Fan

**Affiliations:** ^1^ Department of Radiotherapy and Chemotherapy, Cangzhou Hospital of Integrated Traditional Chinese and Western Medicine, Hebei, Cangzhou, China; ^2^ Department of Radiotherapy and Chemotherapy, Cangzhou Hospital of Integrated Traditional Chinese and Western Medicine East Ward, Hebei, Cangzhou, China

**Keywords:** non-small cell lung cancer, brain metastases, epidermal growth factor receptor, tyrosine kinase inhibitor, craniocerebral radiotherapy

## Abstract

The present study aims to investigate the therapeutic value of third-generation epidermal growth factor receptor (EGFR) tyrosine kinase inhibitors (TKIs) combined with cranial radiotherapy (RT) in patients with EGFR-positive non-small cell lung cancer (NSCLC) and brain metastases (BMs).

**Methodology:**

This is a retrospective study that involved 213 patients with EGFR-NSCLC and BMs, with the patients divided into two groups: the upfront cranial RT (ucRT) group (n = 96) and the non-ucRT group (n = 117). All patients were administered with osimertinib, and those in the ucRT group also underwent RT. The overall survival (OS), progression-free survival (PFS) and intracranial PFS (IPFS) of the two groups were compared.

**Results:**

The ucRT group manifested a markedly higher IPFS than the non-ucRT group (29.65 months vs 21.8 months; P < 0.0001). The subgroup analysis revealed that patients with oligometastases (OLOGO-BMs; 1–3 BMs) demonstrated a notably longer OS (44.5 months vs 37.3 months; P < 0.0001), PFS (32.3 months vs 20.8 months; P = 0.6884) and IPFS (37.8 months vs 22.1 months; P < 0.0001) in the ucRT group than in the non-ucRT group. However, for patients with multiple BMs, there was no significant difference in OS (27.3 months vs 34.4 months; P = 0.0710) and PFS (13.7 months vs 13.2 months; P = 0.0516) between the ucRT group and the non-ucRT group; the ucRT group exhibited a higher IPFS (26.4 months vs 21.35 months; P = 0.0028). Cox’s multivariate analysis of patients with OLOGO-BM indicated that the use of ucRT was linked to a better OS (heart rate [HR] = 0.392; 95% confidence interval [CI]: 0.178–0.863; P = 0.020) and PFS (HR = 0.558; 95% CI: 0.316–0.986; P = 0.044).

**Conclusion:**

Upfront cerebral cranial stereotactic radiosurgery can improve outcomes in EGFR-positive patients with NSCLC and OLOGO-BM. However, for patients with multiple BMs, the preferable strategy may be pre-treatment with EGFR-TKIs.

## 1 Introduction

Brain metastases (BMs) are the most common and debilitating complications linked to non-small cell lung cancer (NSCLC) occurring in an estimated 20%–40% of all diagnosed cases (1). The prevalence is even more alarming in patients who test positive for mutations in the epidermal growth factor receptor (EGFR) gene, with rates soaring between 44% and 63% (2). During the era when chemotherapy was the mainstay of treatment, the effectiveness of chemotherapeutic agents was markedly limited, as these drugs struggled to penetrate the blood–brain barrier. This shortcoming led to a dire prognosis for patients with BMs (3).

The landscape of treatment, however, has been radically transformed by the advent of targeted therapies, particularly small-molecule EGFR tyrosine kinase inhibitors (EGFR-TKIs). These have emerged as the front-line treatment for advanced NSCLC in patients with EGFR mutations, demonstrating far superior blood–brain barrier penetration compared with traditional chemotherapy (4). For instance, a phase II trial revealed that patients with NSCLC and BMs treated with erlotinib as a first-line regimen experienced median overall survival (OS) rates of 15.9–22.9 months and progression-free survival (PFS) rate of 5.8–14.5 months (5). Further clinical trials have demonstrated similar or even improved outcomes, including impressive intracranial PFS (IPFS) (6, 7).

Historically, treatment options for BM were mainly confined to whole-brain radiotherapy (WBRT), stereotactic radiosurgery (SRS) and surgical resection. Emerging evidence suggests a synergistic therapeutic effect between cranial radiotherapy (RT) and EGFR-TKI. The former appears to facilitate the penetration of the latter through the blood–brain barrier, thereby increasing its concentration in the cerebrospinal fluid and amplifying RT’s antitumour effects (8). Numerous studies confirm the potential of combining cranial RT and EGFR-TKI for managing BM, although the findings are not universally consistent (9). However, this enhanced survival often comes at the cost of detrimental side effects, such as memory loss and cognitive decline. These are mainly due to the cranial RT, thereby igniting ongoing debates over its role and timing in patients who are EGFR-positive (10).

Recent advances in third-generation EGFR-TKIs have shown particular promise for patients with NSCLC and BM, owing to their superior penetration through the blood–brain barrier (11). Studies such as the FLAURA and AENEAS trials have shown extended PFS and IPFS with the use of osimertinib and almonertinib, respectively (12, 13). While the efficacy of these third-generation inhibitors is evident, the role of cranial RT in conjunction with these advanced drugs remains relatively unclear. Although there is evidence supporting the enhanced effectiveness of combining first or second-generation EGFR-TKIs with cranial RT (14), questions linger regarding whether such a synergistic benefit extends to third-generation EGFR-TKIs.

Given the scarcity of literature addressing this specific combination in patients with NSCLC and both EGFR-positivity and BMs, the authors of the present paper conducted a retrospective study to explore the clinical significance and optimal timing of cranial RT in this unique patient cohort.

## 2 Materials and methods

### 2.1 Patient selection

The medical records of patients with NSCLC carrying EGFR mutations and diagnosed with BMs admitted to Cangzhou Hospital of Integrative Traditional Chinese and Western Medicine (TCM-WM) of Hebei between January 2018 and December 2022 were meticulously collated. The inclusion criteria were as follows: patients (1) with histopathologically verified lung adenocarcinoma; (2) with BMs, ascertained by magnetic resonance imaging (MRI); (3) with either exon 19 deletion (19DEL) or exon 21 points mutation (21L858R mutation), confirmed by genetic testing; and (4) who received osimertinib as an initial treatment regimen.

The exclusion criteria were as follows: patients (1) who previously used EGFR-TKI without receiving osimertinib during cranial RT or within one week after RT; (2) who underwent surgical resection at an initial BM diagnosis; and (3) with other serious illnesses that could affect their survival or cognitive function, such as stroke, Parkinson’s disease or severe liver or kidney dysfunction.

A total of 213 patients fitting these criteria were recruited for the study, and their baseline characteristics, namely age, gender, Karnofsky performance score (KPS), smoking history, EGFR mutation type, number of BMs, maximum BM diameter, BM symptoms and extracranial metastases, were documented. All the patients were evaluated using the diagnostic-specific grading prognostic score (DS-GPA) for BMs (15). The data collection cut-off date was 1 December 2022. The study received approval from the ethics committee of Cangzhou Hospital of Integrative TCM-WM of Hebei, and all patient-involved procedures complied with the principles of the Declaration of Helsinki. Informed consent was obtained from all the patients.

### 2.2 Treatment protocols

Patients were assigned to either the upfront cranial RT group (ucRT) or the non-RT group based on cranial RT application timing. The ucRT cohort comprised patients receiving cranial RT concurrent with a third-generation EGFR-TKI or patients commencing third-generation EGFR-TKI therapy after undergoing cranial RT (with an interval not exceeding one week). The term ‘upfront’ refers to treatment given as the first-line therapy for patients with BMs before the occurrence of any evidence of intracranial or systemic progression. The non-ucRT group included patients receiving osimertinib as their sole first-line treatment; all the patients were administered osimertinib at a standard daily dose of 80 mg. To reduce neurotoxicity and preserve cognitive function, cranial RT involved hippocampal-avoidance WBRT (HA-WBRT, 30 Gy/10 f; hippocampi doses: Dmax ≤ 17 Gy, Dmean ≤ 10 Gy and D40 ≤ 9 Gy) and and SRS (dose determined in line with the Rt Oncology Group 90-05 and 95-08 institutional guidelines) (16, 17). The former was predominantly used in patients with multiple BMs (BM number > 3) and patients not eligible for SRS (n = 36), whereas SRS was primarily utilised in patients with 1–3 BMs (n = 38).

The RT dose and plan were recorded as follows: Among the 213 patients in this study, 96 had oligometastatic brain disease (ucRT group: n = 47; non-ucRT group: n = 49). All 47 patients in the ucRT group underwent SRS treatment, with the following clinical data:

In patients with 1 BM (n = 18), the median BM diameter was 0.85 cm (0.7–3 cm), with a mean of 1.3 cm. The brain metastasis volume was <1 cm^3^ in 12 patients, and 1.2 cm^3^, 5.9 cm^3^, 9.5 cm^3^, 10.4 cm^3^, 10.8 cm^3^ and 15.8 cm^3^ in the remaining 6 patients, respectively. The SRS dose fractionation scheme was: 21–23 Gy/1 F in the 12 patients with a BM diameter of <1 cm^3^, 18 Gy/1 F in the 1.2 cm^3^ lesion, 27 Gy/3 F in the 5.9 cm^3^ lesion and 30 Gy/5 F in the rest.

In patients with 2 BMs (n = 23), the median BM maximum diameter was 1.5 cm (0.6–2.9 cm), with a mean of 1.6 cm. The brain metastasis volume and the corresponding SRS dose fractionation scheme were: <1 cm^3^ in 19 lesions (20–23 Gy/1 F), 1–5 cm^3^ in 14 lesions (18–20 Gy/1 F or 27–30 Gy/3–5 F), 5–10 cm^3^ in 10 lesions (27–30 Gy/3–5 F) and >10 cm^3^ in 3 lesions (30 Gy/5 F).

In patients with 3 BMs (n = 6), the median BM maximum diameter was 1.2 cm (0.6–2.8 cm), with a mean of 1.5 cm. The brain metastasis volume and the corresponding SRS dose fractionation scheme were: <1 cm^3^ in 10 lesions (22–23 Gy/1 F), 1–5 cm^3^ in 3 lesions (20 Gy/1 F, or 27 Gy/3 F), 5–10 cm^3^ in 2 lesions (27–30 Gy/3–5 F), and >10 cm^3^ in 3 lesions (30 Gy/5 F).

### 2.3 Follow-up and response assessment

For the ucRT group, systemic and intracranial response assessments were undertaken 1 month after RT completion and every 2–3 months subsequently. For the non-ucRT group, tumour response was routinely evaluated every 2–3 months after the commencement of the osimertinib treatment. The cut-off date for the follow-up was December 2022. In cases of suspected intracranial or systemic progression, cranial MRI or computed tomography were swiftly performed. Treatment response was evaluated in accordance with the Response Evaluation Criteria in Solid Tumours version 1.1.

### 2.4 Study endpoints

The primary endpoints comprised OS, PFS and IPFS. The first, OS, was defined as the duration from the initiation of osimertinib treatment to either the patient’s death or the last known follow-up; PFS referred to the period from the commencement of osimertinib treatment to systemic progression, death from any cause or the last known follow-up; and IPFS referred to the time from the onset of osimertinib treatment to intracranial tumour progression, death from any cause or the last known follow-up. All the patients were subject to data review at the final follow-up visit.

### 2.5 Statistical analysis

Data analysis was executed using the SPSS software 27.0 and GraphPad Prism software 8.0. The baseline characteristics of patients in both groups were contrasted using the chi-square test or Fisher’s exact test, and OS, PFS and IPFS were calculated using the Kaplan–Meier method. Differences in survival curves between the two groups were assessed using the log-rank test. The association of various factors with survival was analysed using the Cox proportional risk model. In the univariate model, variables linked with survival (P < 0.10) were incorporated into the multivariate model, with a two-sided P-value of <0.05 denoting statistical significance.

To account for potential selection bias arising from the retrospective nonrandomised study design, differences in OS, PFS and IPFS between the two groups were analysed using inverse probability of treatment weighting (IPTW). The presence or absence of BM symptoms was not included as a covariate in the IPTW, as this factor represents an obvious indication for determining whether to perform cranial RT. All other baseline characteristics were incorporated into the covariates of IPTW.

In the present propensity score matching (PSM) process, the authors utilised a 1:1 nearest-neighbour-matching algorithm with a calliper width of 0.02 to align patients from the ucRT (unconsolidated RT) group with those from the non-ucRT group. This strict matching criterion was employed to ensure a high degree of similarity between the matched pairs in terms of their propensity scores. Despite the authors’ efforts to closely match patients from both groups, the inherent variability within the patient cohorts meant that not all individuals could be paired. This discrepancy in the final matched numbers reflects the stringent calliper setting, which prioritises the quality of the match over the quantity.

## 3 Results

### 3.1 Characteristics of the patient cohort

This study involved a cohort of 213 patients, 96 of whom were placed in the ucRT group, while 117 were allocated to the non-ucRT group. Following IPTW, 74 patients were placed in each group. The baseline attributes of the patients in both groups are outlined **Table 1**. It can be seen that there were no significant disparities between the two groups in terms of age, sex, smoking history, KPS, EGFR mutation type, number of BMs and DS-GPA score. Notably, the ucRT group had a higher prevalence of patients with symptomatic BM (P < 0.05). The median follow-up time was 18.6 months (range: 3.2–48.5 months) for the ucRT group and 16.4 months (range: 2.7–46.3 months) for the non-ucRT group.

**Table 1:** Comparison of general clinical information of patients in the ucRT and non-ucRT groups.

Characteristics	Before matching		X^2^	P	After matching		X^2^	P
	ucRT group N(%)	non-ucRT group N(%)			ucRTgroup N(%)	non-ucRT group N(%)		
Age								
<60	59(61.5)	67(57.3)			44(59.5)	41(55.5)		
≥60	37(38.5)	50(42.7)	0.384	0.536	30(40.5)	33(44.5)	0.249	0.618
Gender								
Male	62(64.6)	69(59.0)			47(63.5)	49(66.2)		
Female	34(35.4)	48(41.0)	0.701	0.403	27(36.5)	25(33.8)	0.119	0.731
KPS								
<80	22(22.9)	28(23.9)			17(23.0)	18(24.3)		
≥80	74(77.1)	89(76.1)	0.030	0.862	57(77.0)	56(75.7)	0.037	0.847
Smoking history								
Yes	31(32.3)	33(28.2)			22(29.7)	22(29.7)		
No	65(67.7)	84(71.8)	0.419	0.517	52(70.3)	52(70.3)	0.000	1.000
EGFR mutations								
19 deletion	50(52.1)	57(48.7)			35(47.3)	38(51.4)		
L858R mutations	46(47.9)	60(51.3)	0.239	0.625	39(52.7)	36(48.6)	0.243	0.622
BM symptoms								
Yes	53(55.2)	24(20.5)			36(48.6)	21(28.4)		
No	43(44.8)	93(79.5)	27.501	<0.001	38(51.4)	53(71.6)	6.420	0.011
BM number								
1-3	47(49.0)	49(41.9)			38(51.4)	29(39.2)		
>3	49(51.0)	68(58.1)	1.067	0.302	36(48.6)	45(60.8)	2.735	0.098
Maximum diameter of BM								
≤10mm	35(36.5)	56(47.9)			34(45.9)	28(37.8)		
>10mm	61(63.5)	61(52.1)	2.803	0.094	40(54.1)	46(62.2)	0.999	0.317
Extracranial metastasis								
Yes	50(52.1)	69(59.0)			40(54.1)	40(54.1)		
No	46(47.9)	48(41.0)	1.016	0.314	34(45.9)	34(45.9)	0.000	1.000
DS-GPA score								
1.0-2.0	29(30.2)	29(24.8)			23(31.1)	17(23.0)		
2.5-3.0	33(34.4)	51(43.6)			25(33.8)	31(41.9)		
3.5-4.0	34(35.4)	37(31.6)	1.932	0.381	26(35.1)	26(35.1)	1.543	0.462

KPS, karnofsky score; EGFR, epidermal growth factor receptor; BM, brain metastasis; DS-GPA, diagnostic specific grading prognostic score.

### 3.2 Survival analysis

**Figure 1:**
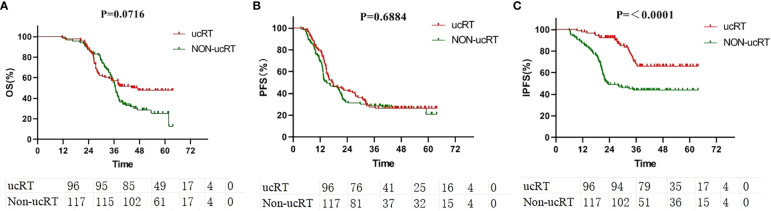
**(A)** Comparison of OS in the ucRT and non-ucRT groups. **(B)** Comparison of PFS in the ucRT and non-ucRT groups. **(C)** Comparison of IPFS in the ucRT and non-ucRT groups.

For the entire cohort, the median OS was 36.4 months. The ucRT and non-ucRT cohorts had a median OS of 37.6 and 36.2 months, respectively (P = 0.0716; **Figure 1A**). In terms of PFS, the median for all patients was 16.2 months, with the ucRT and non-ucRT groups demonstrating 17.35 and 14.4 months, respectively (P = 0.6884; **Figure 1B**). As for IPFS, the overall median was 27.3 months, with the ucRT and non-ucRT groups reporting 29.65 and 21.8 months, respectively (P < 0.0001; **Figure 1C**).

### 3.3 Survival analysis in the inverse probability of the treatment weighting cohort

**Figure 2:**
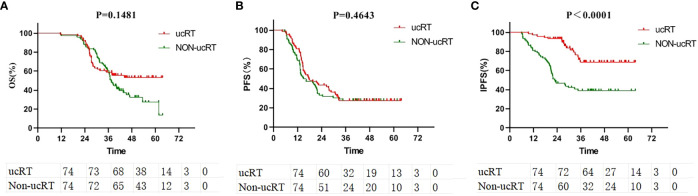
**(A)** Comparison of OS between ucRT and non-ucRT groups after IPTW. **(B)** Comparison of PFS between ucRT and non-ucRT groups after IPTW. **(C)** Comparison of IPFS in ucRT and non-ucRT groups after IPTW.

All the patients underwent IPTW, after which survival analysis was conducted on 74 patients in each group. Here, age, gender, smoking history, KPS, EGFR mutation type, number of BMs, maximum BM diameter and extracranial metastasis status were balanced. After IPTW, there were no significant deviations in OS and PFS between the two groups (P = 0.1481 and P = 0.4643; **Figures 2A, B**). However, IPFS was markedly higher in the ucRT group than in the non-ucRT group (P < 0.0001; **Figure 2C**).

### 3.4 Subgroup survival analysis stratified by the brain metastases number

**Figure 3:**
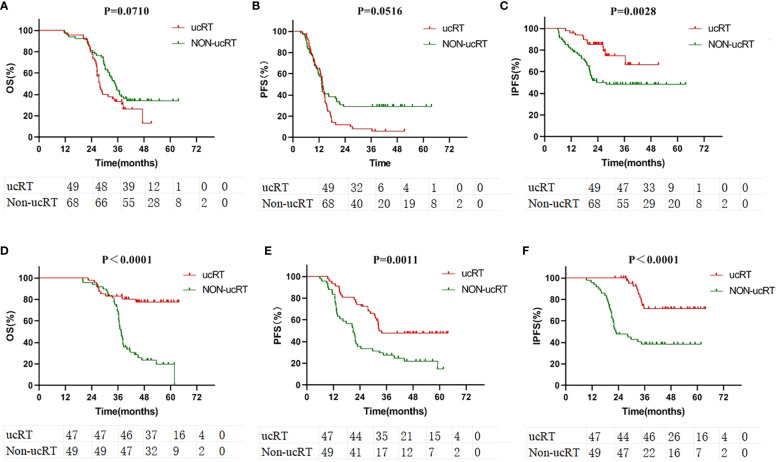
**(A)** Comparison of OS in the subgroup of patients with multiple brain metastases. **(B)** Comparison of PFS in the subgroup of patients with multiple brain metastases. **(C)** Comparison of IPFS in the subgroup of patients with multiple brain metastases. **(D)** Comparison of OS in the subgroup of patients with oligometastases. **(E)** Comparison of PFS in the subgroup of patients with oligometastases. **(F)** Comparison of IPFS in the subgroup of patients with oligometastases.

The patients were segregated into the multiple-BMs subgroup and the oligometastases (OLOGO-BMs) subgroup based on the number of BMs, with OLOGO-BM characterised as 1–3 BM lesions with a maximum diameter of ≤3.5 cm (maximum diameter of ≤3.0 cm in the ucRT group and a maximum diameter of ≤3.5 cm in the non-ucRT group) (18). The remaining cases were classified as having multiple BMs. Survival analysis was then performed on patients from both groups; in the multiple-BMs subgroup before IPTW the ucRT and non-ucRT groups demonstrated a median OS of 27.3 and 34.35 months, respectively (P = 0.0710; **Figure 3A**); a median PFS of 13.7 and 13.15 months, respectively (P = 0.0516; **Figure 3B**); and a median IPFS of 26.4 and 21.35 months, respectively (P = 0.0028; **Figure 3C**). Meanwhile, in the OLOGO-BMs subgroup, the ucRT and non-ucRT groups had a median OS of 44.5 and 37.3 months, respectively (P < 0.0001; **Figure 3D**); a median PFS of 32.3 and 20.8 months, respectively (P = 0.0011; **Figure 3E**); and a median IPFS of 37.8 and 22.1 months, respectively (P < 0.0001; **Figure 3F**). Furthermore, the Cox multifactorial analysis of OS and PFS in the OLOGO-BMs subgroup demonstrated that ucRT was linked with better OS (heart rate [HR] = 0.392; 95% confidence interval [CI]: 0.178–0.863; P = 0.020) and PFS (HR = 0.558; 95% CI: 0.316–0.986; P = 0.044) than seen in the non-ucRT group (**Supplementary Tables 1**, **2**). These findings validate the effectiveness of ucRT in patients with OLOGO-BM.

**Figure 4:**
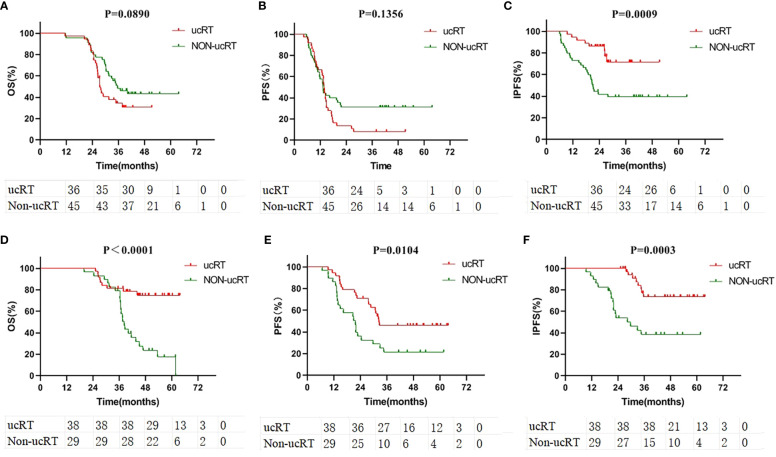
**(A)** Comparison of OS in the subgroup of patients with multiple brain metastases after IPTW. **(B)** Comparison of PFS in the subgroup of patients with multiple brain metastases after IPTW. **(C)** Comparison of IPFS in the subgroup of patients with multiple brain metastases after IPTW. **(D)** Comparison of OS in the subgroup of patients with oligometastases after IPTW. **(E)** Comparison of PFS in the subgroup of patients with oligometastases after IPTW. **(F)** Comparison of IPFS in the subgroup of patients with oligometastases after IPTW.

**Figure 5:**
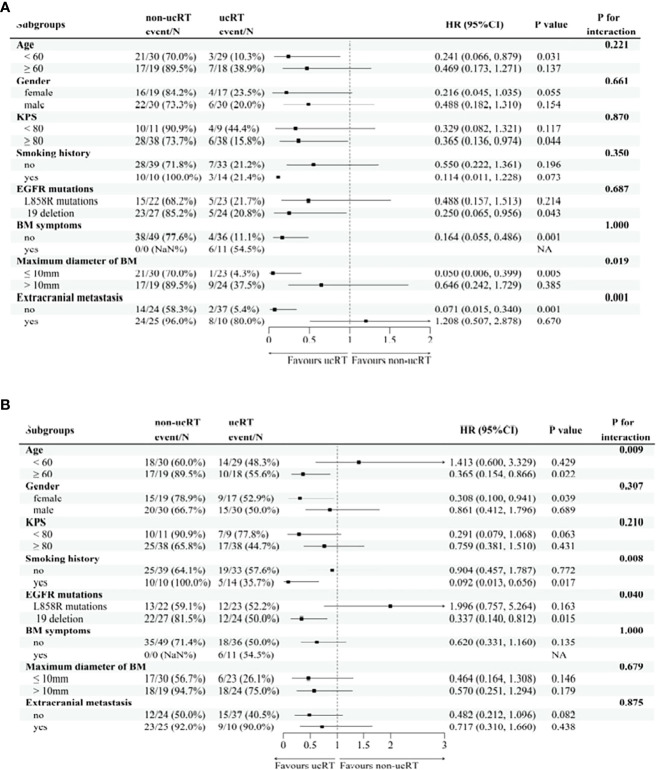
**(A)** the subgroup of forest map for Overall Survival in patients with oligocerebral metastasis. **(B)** the subgroup of forest map for Progression-Free Survival in patients with oligocerebral metastasis.

Following IPTW, 38 patients in the ucRT group and 29 patients in the non-ucRT group were successfully matched in the OLOGO-BM subgroup. In the multiple-BMs subgroup, 36 patients in the ucRT group were successfully matched with 45 patients in the non-ucRT group. The discrepancies in covariates between the two groups are outlined in **Table 2**. In the OLOGO-BM subgroup, all 38 patients in the ucRT group received SRS. Conversely, in the multiple-BMs subgroup, all 36 patients in the ucRT group underwent HA-WBRT. There were no significant post-IPTW disparities in OS and PFS between the two groups in the multiple-BMs subgroup (27.3 months and 34.9 months; P = 0.0890; **Figure 4A**; 13.7 months and 13.2 months; P = 0.1356; **Figure 4B**). Notably, IPFS was significantly higher in the ucRT group than in the non-ucRT group, with medians of 26.35 and 21.0 months, respectively (P = 0.0009; **Figure 4C**). In the OLOGO-BM subgroup, post-IPTW, the ucRT group reported a significantly higher OS, PFS and IPFS compared with the non-ucRT group, with a median OS of 44.45 and 37.5 months, respectively (P < 0.0001; **Figure 4D**); a median PFS of 31.95 and 20.8 months, respectively (P = 0.0104; **Figure 4E**); and a median IPFS of 37.7 and 24.3 months, respectively (P = 0.0003; **Figure 4F**). As can be seen from the forest map of OS and PFS of patients with oligocerebral metastasis, extracranial metastases and patients with maximum BM diameter > 10mm can benefit from non-ucRT in terms of OS. Patients with age < 60 and L858R mutation may have prolonged PFS from non-ucRT (**Figure 5**).

**Table 2:** Comparison of general clinical information of patients in the subgroup of oligometastases and the subgroup of multiple brain metastases after IPTW.

Characteristics	Oligometastases subgroup		X^2^	P	Multiple brain metastases subgroup		X^2^	P
	ucRT group N(%)	non-ucRT group N(%)			ucRT group N(%)	non-ucRT group N(%)		
Age								
<60	23(60.5)	16(55.2)			21(58.3)	25(55.6)		
≥60	15(39.5)	13(44.8)	0.194	0.660	15(41.7)	20(44.4)	0.063	0.802
Gender								
Male	24(63.2)	20(69.0)			23(63.9)	29(64.4)		
Female	14(36.8)	9(31.0)	0.246	0.620	13(36.1)	16(35.6)	0.003	0.959
KPS								
<80	7(18.4)	7(24.1)			10(27.8)	11(24.4)		
≥80	31(81.6)	22(75.9)	0.325	0.568	26(72.2)	34(75.6)	0.116	0.734
Smoking history								
Yes	11(28.9)	7(24.1)			11(30.6)	15(33.3)		
No	27(71.1)	22(75.9)	0.194	0.660	25(69.4)	30(66.7)	0.071	0.790
EGFR mutations								
19 deletion	19(50.0)	18(62.1)			16(44.4)	20(44.4)		
L858R mutations	19(50.0)	11(37.9)	0.969	0.325	20(55.6)	25(55.6)	0.000	1.000
BM symptoms								
Yes	6(15.8)	0(0.0)			30(83.3)	21(46.7)		
No	32(84.2)	29(100.0)	*	0.012	6(16.7)	24(53.3)	11.531	<0.001
BM number								
1-3	38(100.0)	29(100.0)			0(0.0)	0(0.0)		
>3	0(0.0)	0(0.0)	*	*	36(100.0)	45(100.0)	*	*
Maximum diameter of BM								
≤10mm	22(57.9)	14(48.3)			12(33.3)	14(31.1)		
>10mm	16(42.1)	15(51.7)	0.612	0.434	24(66.7)	31(68.9)	0.045	0.831
Extracranial metastasis								
Yes	9(23.7)	13(44.8)			31(86.1)	31(68.9)		
No	29(76.3)	16(55.2)	3.334	0.068	5(13.9)	14(31.1)	3.304	0.069
DS-GPA score								
1.0-2.0	1(2.6)	3(10.3)			22(61.1)	17(37.8)		
2.5-3.0	14(36.9)	14(48.3)			11(30.6)	17(37.8)		
3.5-4.0	23(60.5)	12(41.4)	3.216	0.212	3(8.3)	11(24.4)	5.567	0.062

KPS, karnofsky score; EGFR, epidermal growth factor receptor; BM, brain metastasis; DS-GPA, diagnostic specific grading prognostic score; * indicates that no statistical analysis was performed.

### 3.5 Failure modes

**Figure 6:**
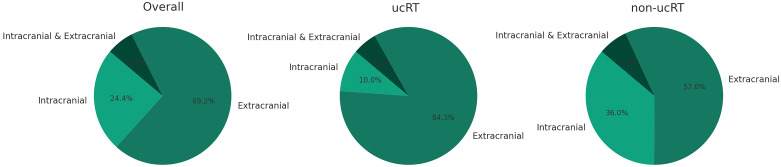
Patterns of Tumor Progression in Patients with and without uCRT.

Throughout the follow-up period, 156 patients (73.2%) registered either intracranial or extracranial tumour progression. Of these, the ucRT group exhibited a predominant first site of progression extracranially, whereas the non-ucRT group showed a significant trend towards intracranial progression as the initial site (P < 0.001; **Figure 6**).

### 3.6 Adverse events

The EGFR-TKI-related adverse reactions: The incidence of adverse reactions in the ucRT group and the non-ucRT group was 75.0% and 73.5%, respectively (P = 0.804). Among them, diarrhoea was the most common (45.8% vs 44.4%, P = 0.839), followed by rashes (44.8% vs 43.6%, P = 0.860), paronychia (32.3% vs 30.8%, P = 0.812), dry skin (31.3% vs 29.9%, P = 0.833), etc. Most were grade 1–2 (97.9% vs 98.3%, P = 1.000; **Supplementary Table 3**). Adverse reactions related to brain RT: The incidence of adverse reactions was 51.3% and 85.7% in the OLOGO-BM group and multiple-BMs groups, respectively (P < 0.001). The most common adverse reactions were dizziness (31.9% vs 53.1%, P = 0.036), headache (27.7% vs 51.1%, P = 0.019), radiation dermatitis (17.0% vs 47.3%, P = 0.002) and neurocognitive dysfunction (32% vs 85.7%, P < 0.001). The incidence of grade 3 and above adverse reactions was 2.1% and 4.1% (P = 1.000; **Supplementary Table 4**).

## 4 Discussion

As a first-line treatment for stage IV NSCLC with EGFR mutations, osimertinib has substantially extended both OS and PFS compared with first-generation EGFR-TKIs (19). Specifically, in the context of EGFR-positive NSCLC with concurrent BM, third-generation EGFR-TKIs have exhibited remarkable efficacy. One clinical study comparing osimertinib to gefitinib/erlotinib revealed a strikingly extended PFS (15.2 months vs 9.6 months) (20). Additionally, the FLAURA clinical trial demonstrated significant enhancements in IPFS for the osimertinib group as opposed to the gefitinib/erlotinib group (21). Similar outcomes were reported in domestic studies involving other third-generation EGFR-TKIs, such as almonertinib and furmonertinib (13, 22).

While RT remains an essential treatment option for patients with BM, particularly those manifesting symptoms, the introduction of highly effective third-generation EGFR-TKIs has stirred debate concerning the role and optimal timing of cranial RT. Several studies have tried to evaluate the effectiveness of combining first/second-generation EGFR-TKIs with cranial RT versus monotherapy but have yielded inconclusive results (9, 23–25). These disparate findings point to the ongoing ambiguity around the ideal timing for cranial RT in the era of potent EGFR-TKIs.

The present analysis was inspired by previous research aiming to discern whether a synergy exists between pre-emptive cranial RT and third-generation EGFR-TKIs in prolonging survival. Although existing studies suggest that the addition of cranial RT to third-generation EGFR-TKI treatments does not dramatically extend OS and PFS, these results should be interpreted cautiously. Most of the patients in these studies were administered third-generation EGFR-TKIs as a second-line treatment or beyond, which may have introduced bias (26–28).

In the present study, which exclusively involves patients treated with osimertinib as a first-line option, the authors found no significant differences in OS and PFS between the ucRT group and the non-ucRT group. However, IPFS was notably higher in the ucRT group than in the non-ucRT group, especially among patients with limited BM (1–3 lesions) (29, 30). The subgroup analysis further revealed that ucRT could be associated with improved OS and PFS, especially in patients with OLOGO-BM. This improvement may be attributed to the heightened radiosensitivity of EGFR-positive NSCLC cells and the ability of cranial RT to efficiently clear intracranial tumours while potentially facilitating better EGFR-TKI penetration through the blood–brain barrier.

It is important to note that the present study is retrospective and carries inherent limitations, such as a risk of selection bias, a lack of exploration of the role of cranial RT before and after intracranial progression during osimertinib treatment and a failure to assess the adverse effects of different RT modalities. Furthermore, inconsistencies in follow-up treatment choices among treating physicians may have also affected the outcomes. Nonetheless, the present findings contribute valuable insights into a heavily debated and clinically significant issue, warranting further prospective trials for confirmation.

## 5 Conclusion

For patients with EGFR-positive NSCLC and BM, stratification according to BM status can guide treatment selection. For patients with OLOGO-BM, ucSRS may improve OS, PFS and IPFS more effectively than ucEGFR-TKI, advocating for early cranial SRS for eligible patients who can tolerate SRS. Conversely, for patients with multiple BMs, ucRT may not be as effective as upfront EGFR-TKI in improving OS and PFS and may increase neurotoxicity and reduce patients’ quality of life. Thus, upfront EGFR-TKI treatment might be the optimal choice for patients with multiple BMs. These conclusions warrant further validation through prospective studies with larger sample sizes.

## Data availability statement

The original contributions presented in the study are included in the article/**Supplementary Material**. Further inquiries can be directed to the corresponding author.

## Ethics statement

The studies involving humans were approved by Ethics Committee of Cangzhou Hospital of Integrative TCM-WM of Hebei. The studies were conducted in accordance with the local legislation and institutional requirements. The participants provided their written informed consent to participate in this study.

## Author contributions

JZ: Conceptualization, Formal analysis, Resources, Writing – original draft. YZ: Conceptualization, Data curation, Investigation, Methodology, Writing – original draft. YS: Conceptualization, Data curation, Formal analysis, Methodology, Writing – review & editing. LX: Data curation, Formal analysis, Methodology, Project administration, Writing – original draft. HL: Data curation, Resources, Validation, Writing – original draft. XY: Data curation, Investigation, Writing – original draft. KF: Investigation, Software, Supervision, Writing – original draft.
