# Supplementary material for: The efficacy of upfront craniocerebral radiotherapy and epidermal growth factor receptor-tyrosine kinase inhibitors in patients with epidermal growth factor receptor-positive non-small cell lung cancer with brain metastases
Source: Front Oncol. 2024 Jan 19;13:1259880. doi: 10.3389/fonc.2023.1259880 (PMC10834619; doi:10.3389/fonc.2023.1259880)
Supplement: Supplementary file 1 [file Table_1.docx]

Supplementary Table 1 Univariate and multivariate analysis of OS in the subgroup of patients with oligometastases

|  | Univariate analysis | | | Multivariate analysis | | |
| --- | --- | --- | --- | --- | --- | --- |
| Characteristics | HR | 95%CI | P | HR | 95%CI | P |
| Pretreatment |  |  |  |  |  |  |
| non-ucRT VS ucRT | 0.206 | 0.102-0.415 | ＜0.001 | 0.392 | 0.178-0.863 | 0.020 |
| Age |  |  |  |  |  |  |
| ＜60 VS ≥60 | 2.111 | 1.190-3.747 | 0.011 | 2.873 | 1.352-6.106 | 0.006 |
| Gender |  |  |  |  |  |  |
| Female VS Male | 0.657 | 0.369-1.171 | 0.154 |  |  |  |
| KPS |  |  |  |  |  |  |
| ＜80 VS ≥80 | 0.435 | 0.232-0.816 | 0.010 | 0.443 | 0.155-1.265 | 0.128 |
| Smoking history |  |  |  |  |  |  |
| No VS Yes | 1.241 | 0.654-2.352 | 0.509 |  |  |  |
| EGFR mutations |  |  |  |  |  |  |
| L858R mutations VS 19 deletion | 1.394 | 0.784-2.478 | 0.258 |  |  |  |
| BM symptoms |  |  |  |  |  |  |
| No VS Yes | 1.533 | 0.649-3.620 | 0.330 |  |  |  |
| Maximum diameter of BM |  |  |  |  |  |  |
| ≤10mm VS ＞10mm | 1.996 | 1.116-3.571 | 0.020 | 0.887 | 0.460-1.711 | 0.720 |
| Extracranial metastasis |  |  |  |  |  |  |
| No VS Yes | 20.437 | 8.968-46.571 | ＜0.001 | 22.473 | 6.925-72.929 | ＜0.001 |
|  |  |  |  |  |  |  |
|  |  |  |  |  |  |  |

Notes: KPS: karnofsky score; EGFR: epidermal growth factor receptor; BM: brain metastasis;

Supplementary Table 2 Univariate and multivariate analysis of PFS in the subgroup of patients with oligometastases

|  | Univariate analysis | | | Multivariate analysis | | |
| --- | --- | --- | --- | --- | --- | --- |
| Characteristics | HR | 95%CI | P | HR | 95%CI | P |
| Pretreatment |  |  |  |  |  |  |
| non-ucRT VS ucRT | 0.436 | 0.260-0.729 | 0.002 | 0.558 | 0.316-0.986 | 0.044 |
| Age |  |  |  |  |  |  |
| ＜60 VS ≥60 | 1.578 | 0.953-2.613 | 0.076 | 1.361 | 0.700-2.644 | 0.364 |
| Gender |  |  |  |  |  |  |
| Female VS Male | 0.682 | 0.410-1.135 | 0.141 |  |  |  |
| KPS |  |  |  |  |  |  |
| ＜80 VS ≥80 | 0.452 | 0.256-0.796 | 0.006 | 0.311 | 0.114-0.849 | 0.023 |
| Smoking history |  |  |  |  |  |  |
| No VS Yes | 1.033 | 0.577-1.849 | 0.912 |  |  |  |
| EGFR mutations |  |  |  |  |  |  |
| L858R mutations VS 19 deletion | 1.368 | 0.828-2.259 | 0.221 |  |  |  |
| BM symptoms |  |  |  |  |  |  |
| No VS Yes | 0.920 | 0.396-2.139 | 0.847 |  |  |  |
| Maximum diameter of BM |  |  |  |  |  |  |
| ≤10mm VS ＞10mm | 2.495 | 1.494-4.168 | ＜0.001 | 1.677 | 0.948-2.969 | 0.076 |
| Extracranial metastasis |  |  |  |  |  |  |
| No VS Yes | 9.700 | 5.392-17.449 | ＜0.001 | 10.242 | 3.873-27.087 | ＜0.001 |
|  |  |  |  |  |  |  |
|  |  |  |  |  |  |  |

Notes: KPS: karnofsky score; EGFR: epidermal growth factor receptor; BM: brain metastasis;

Supplementary Table 3 Comparison of the EGFR-TKIs related adverse events of the ucRT and non-ucRT groups

| Adverse event | ucRT group N(%) | non-ucRT group N(%) | P |
| --- | --- | --- | --- |
| Occurrence of adverse events |  |  |  |
| Yes | 72(75.0) | 86(73.5) |  |
| No | 24(25.0) | 31(26.5) | 0.804 |
| Diarrhea |  |  |  |
| Yes | 44(45.8) | 52(44.4) |  |
| No | 52(54.2) | 65(55.6) | 0.839 |
| Erythra |  |  |  |
| Yes | 43(44.8) | 51(43.6) |  |
| No | 53(55.2) | 66(56.4) | 0.860 |
| Paronychia |  |  |  |
| Yes | 31(32.3) | 36(30.8) |  |
| No | 65(67.7) | 81(69.2) | 0.812 |
| Dry skin |  |  |  |
| Yes | 30(31.3) | 35(29.9) |  |
| No | 66(68.7) | 82(70.1) | 0.833 |
| Level of adverse events |  |  |  |
| 1-2 grade | 94(97.9) | 115(98.3) |  |
| ≥3 grade | 2(2.1) | 2(1.7) | 1.000 |

| Adverse event | OLOGO-BM group N(%) | multiple-BM group N(%) | P |
| --- | --- | --- | --- |
| Occurrence of adverse events |  |  |  |
| Yes | 24(51.3) | 42(85.7) |  |
| No | 23(48.7) | 7(14.3) | ＜0.001 |
| Dizzy giddy |  |  |  |
| Yes | 15(31.9) | 26(53.1) |  |
| No | 32(68.1) | 23(46.9) | 0.036 |
| Headache |  |  |  |
| Yes | 13(27.7) | 25(51.1) |  |
| No | 34(72.3) | 24(48.9) | 0.019 |
| Radiodermatitis |  |  |  |
| Yes | 8(17.0) | 23(47.3) |  |
| No | 39(83.0) | 26(52.7) | 0.002 |
| Neurocognitive dysfunction |  |  |  |
| Yes | 15(32.0) | 42(85.7) |  |
| No | 32(68.0) | 7(14.3) | ＜0.001 |
| Level of adverse events |  |  |  |
| 1-2 grade | 46(97.9) | 47(95.9) |  |
| ≥3 grade | 1(2.1) | 2(4.1) | 1.000 |

Supplementary Table 4 Comparison of the radiation-related adverse events of the OLOGO-BM and multiple-BM groups
